# Constructing RBAC Based Security Model in u-Healthcare Service Platform

**DOI:** 10.1155/2015/937914

**Published:** 2015-01-27

**Authors:** Moon Sun Shin, Heung Seok Jeon, Yong Wan Ju, Bum Ju Lee, Seon-Phil Jeong

**Affiliations:** ^1^Department of Computer Engineering, Konkuk University, Chungju, Chungbuk 380-701, Republic of Korea; ^2^Division of Industry Development, Korea Internet & Security Agency, Seoul 138-950, Republic of Korea; ^3^Division of Constitutional Medicine Research, Korea Institute of Oriental Medicine, Deajeon 305-811, Republic of Korea; ^4^Division of Technology and Science, BNU-HKBU United International College, Zhuhai, Guangdong 519085, China

## Abstract

In today's era of aging society, people want to handle personal health care by themselves in everyday life. In particular, the evolution of medical and IT convergence technology and mobile smart devices has made it possible for people to gather information on their health status anytime and anywhere easily using biometric information acquisition devices. Healthcare information systems can contribute to the improvement of the nation's healthcare quality and the reduction of related cost. However, there are no perfect security models or mechanisms for healthcare service applications, and privacy information can therefore be leaked. In this paper, we examine security requirements related to privacy protection in u-healthcare service and propose an extended RBAC based security model. We propose and design u-healthcare service integration platform (u-HCSIP) applying RBAC security model. The proposed u-HCSIP performs four main functions: storing and exchanging personal health records (PHR), recommending meals and exercise, buying/selling private health information or experience, and managing personal health data using smart devices.

## 1. Introduction

At the time of the entry of an aging society, people are interested in health and desire to manage their healthy life by themselves. In particular, with the evolution of medical and IT convergence technologies and mobile smart devices, it is now possible for people to gather information on their health status anytime and anywhere easily using biometric information acquisition devices. Web-based healthcare services, such as Google Health, Microsoft's Health Services Platform (CHF), and PatientLikeMe, can easily help patients with similar diseases using social networking (SNS) and have capability of exchanging information and build a wide range of services. In addition, the size of the healthcare application market is increasingly growing due to the spreading use of smart mobile devices.

u-health based on IT and Health will be the solution for improving quality of life and reducing healthcare cost. Healthcare information systems are largely viewed as the single most important factor in improving nation's healthcare quality and reducing related costs. According to a recent RAND study, the USA could potentially save $81B annually by moving to a universal electronic health record (EHR) system. Not surprisingly, recent government initiatives have pushed for wide-scale adoption of universal EHR by 2014 [1].

And the paradigm of medical treatment health care has changed to the preventive health care. Information security and privacy in the healthcare sector are issues of growing importance. The adoption of digital patient records, the increased regulation, the provider consolidation, and the increasing need for information exchange between patients, providers, and payers all point towards the need for better information security [1].

To support more secure healthcare services, it is necessary to construct security model that can be applied to a personalized healthcare service platform in order to preserve the privacy and protect the personal health records. This should also be a prerequisite of personal healthcare services.

In this paper, we examine security requirements related to privacy protection in u-healthcare service integration platform (u-HCSIP) and extend an RBAC security model. An RBAC, which has many benefits such as usability, expressiveness, and appliance in various environments, can be applied to construct a security model for secure healthcare services, because many users, permissions, various constraints, and a lot of roles are included in u-HCSIP. It is possible to specify the security requirement specifications using an extended RBAC and its components for the complicated environment of u-HCSIP. The proposed u-HCSIP supports a healthy lifestyle by measuring personal health information in a hospital clinical, imaging, and drug data, as well as health information obtained from smart devices. Anyone can enter their daily health-related data such as food consumption, sleeping time, mood, movement, and exercise, allowing them to manage their personal health information of modern smart features.

The rest of the paper is organized as follows.  Section 2 describes previous related works and Section 3 presents potential security threats and specifies security requirements in healthcare service platform. In Section 4, we propose the framework of u-HCSIP and construct a security model using RBAC for privacy protection in u-HCSIP. We also present the design of the software architecture of the u-HCSIP applying RBAC. Finally, Section 5 provides some brief concluding remarks.

## 2. Research Background

**Figure 1:**
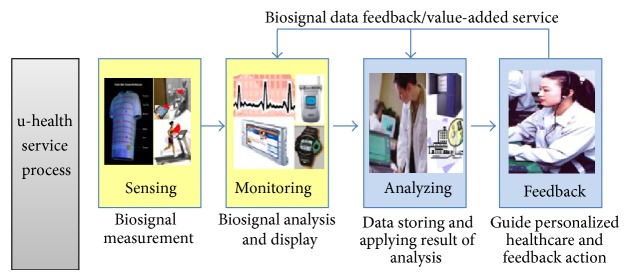
General process of u-health service.

The general process of the u-healthcare service is shown in Figure 1. u-healthcare can be classified as three parts such as u-Medical, u-Silver, and u-Wellness.

These days, according to an aging society, people have more interested in u-Wellness life style. Personalized health care service is needed in u-Wellness. Personal health care services using telemedicine technology can provide users with no restriction in either time or location. In particular, with the development of medical and IT convergence technologies in our increasingly aging society, the consumption and concern for healthy living life have increased. In addition, the development of IT convergence technology makes it possible for users to check their health condition using a smart biodevice to collect their personal health data. With the recent acceleration in the use of various smart devices, the market of mobile health care applications is also growing. According to a survey of global data, the mobile health care market is expected to grow to $8 billion by 2018 [2, 3].

Through the advent of medical technology, doctors are now able to treat their patients more efficiently. For example, doctors can show a treatment or recovery process to the patient by showing them their health conditions via an application. In addition, patients can use an application to communicate with their doctor, allowing them to send or receive medical information and the answers to their questions on their health status.

**Figure 2:**
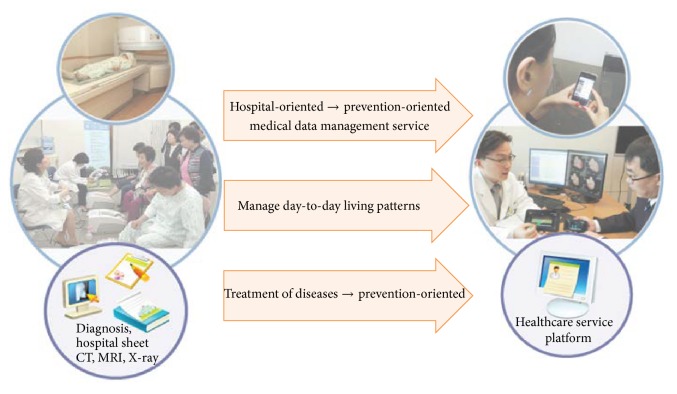
Patient-oriented health data management service.

Currently, u-healthcare service data are stored in the database of the hospital information system, and the patients pay a fee every time they want access to their data from the hospital. When they need to submit their records to another hospital they must have their data put on a CD, or receive their medical records in video data form. However, there exist difficulties to exchange data and to maintain the data among the hospitals [4]. These days, people often eat too much fast food or suffer from stress, so it might lead to an increase in chronic diseases. Thus, the needs for medical services in everyday life are growing rapidly. In particular, the paradigm of medical services in the medical centers for chronic disease has changed to around the general home care. First, daily health-related information (the amount of food consumed and types of exercise, such as momentum) can be entered into a smart device. It is therefore possible to provide customized information on certain health and spiritual guides for diet and exercise needed throughout the day by suing a smart device. This can be of great help in achieving a healthy lifestyle. If users were able to optimize healthcare using the smart devices like tablet PC, smart phones, and the iPad they already have owned, they could achieve a smart life. Because of that reason, many global companies have developed healthcare systems and devices as well as u-healthcare solutions.  Figure 2 shows the needs of a patient-centered care service platform.  Table 1 shows the trend of global companies regarding u-healthcare.

**Figure 3:**
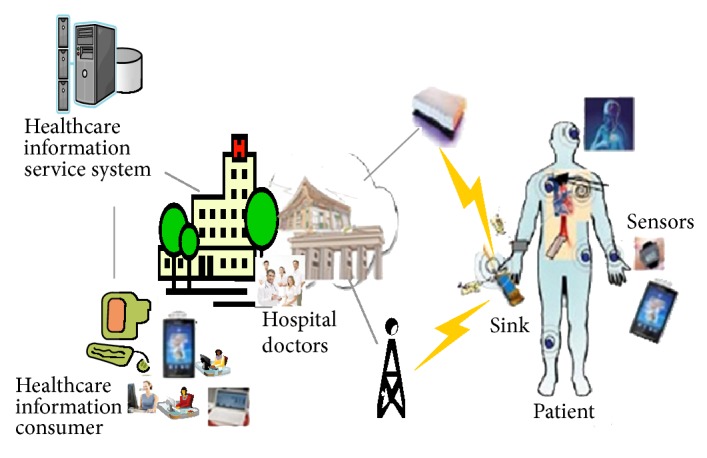
Components of u-healthcare service.

Figure 3 shows the components of the u-healthcare Service and it means health care information consumers are increasing and sensor and biomedical devices are adopted.

In this paper, we suggest moving the focus on hospital-based medical data management to patient-based medical data management, especially for the personalized wellness heath care service through the everyday life.

The patient-oriented medical data management service platform is a healthcare service platform that matches the Health Care 3.0 era to meet the needs of the construction of smart healthcare service platform. The platform comprehensively performs to cover both health data of the hospital or clinic and user health related data measured using smart device like sleep, emotion, exercise, food, and movement data. Further, it can cover to provide health information not only to their own medical staff and specific hospital information system under the patient's consent but also to patients and general users.

**Figure 4:**
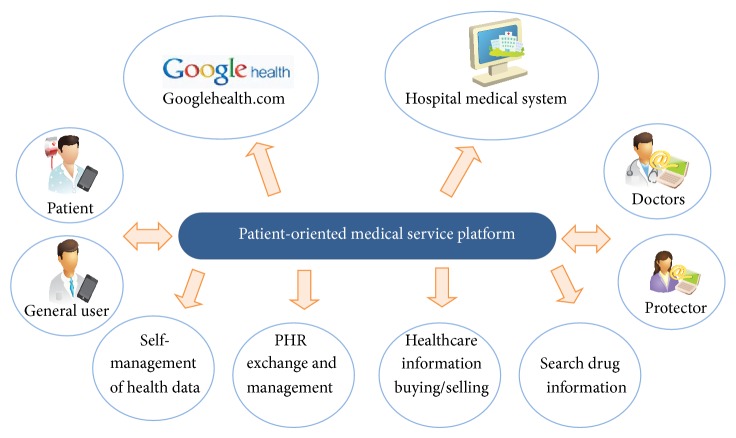
Concept diagram of patient-oriented health data management service.

Patient or personal health data management services should include four main functions: self-management of health data, PHR information exchanges, selling and buying of medical information, and acquisition of drug information.  Figure 4 shows the managing of personal health information using a smart device, providing health information such as effective patient treatment or various cures, and storing and managing video data and medical records in the hospital information system. When utilizing personalized patient-centered healthcare service platform, the view point of the medical service is extended from the medical center to a living area such as a home, fitness center, or business center (e.g., Diabetes using a mobile phone, ECG, blood pressure management). It can reduce repeated visits to the hospital and save in national healthcare costs. Therefore, personal healthcare service platform to support the whole management of personal health data using the smart mobile devices is necessary to customize day life healthcare.

The privacy and security rule of HIPAA requires covered entities to ensure implementation of administrative safeguards in the form of policies, personnel, and physical safeguards to their information infrastructure, and technical safeguards to monitor and control intra- and interorganizational information access. As personal health information is digitized, transmitted, and mined for effective care provision, new threats to patients' privacy are becoming evident [1].

Unauthorized disclosure of health information can have serious consequences including refusal of prospective employment, difficulties in obtaining or continuing insurance contracts and loans, ostracization from family and community groups, and personal embarrassment [1].

A key challenge that attends the adoption of electronic health information networks is therefore to ensure that the principle of consent is meaningfully respected and enforced in electronic contexts [5].

In particular, privacy protection and access control are essential security features in u-healthcare service, so they must be supported. In next section we examine potential vulnerabilities in u-healthcare service system. We analyze security threats to u-healthcare in next section.

## 3. Security Threats against u-Healthcare Services

u-healthcare service was transformed into (health care 2.0) life extension in the treatment of disease services through ICT technology for the (health care 1.0) infection prevention existing in the development of IT fusion technology. Currently, through disease prevention and healthy lifestyle management, the development of health care 3.0 is being pursued. The important keywords of health care 3.0 are miniaturization, day-to-day management, individual customization, patient-oriented care, diagnosis, and treatment [6]. Day-to-day management means developing around preventive medicine from hospital treatment centers. Individual customization means changing from a standard prescription that does not take into account the individual characteristics of personalized treatment regarding genetic factors and temperament. In addition, miniaturization indicates the possibility of early diagnosis by improving the accuracy of the diagnostic treatment. Finally, patient-centered care means development for maximizing the convenience and utility of the patient. Unlike existing hospital databases that store basic patient information, it stores the life patterns of everyday life such as sleep, diet, exercise, and emotional changes. In addition, it provides personalized health care utilizing an analysis of large volumes of data mining techniques such as pattern recognition and classification for an analysis of similar patient diseases.

**Figure 5:**
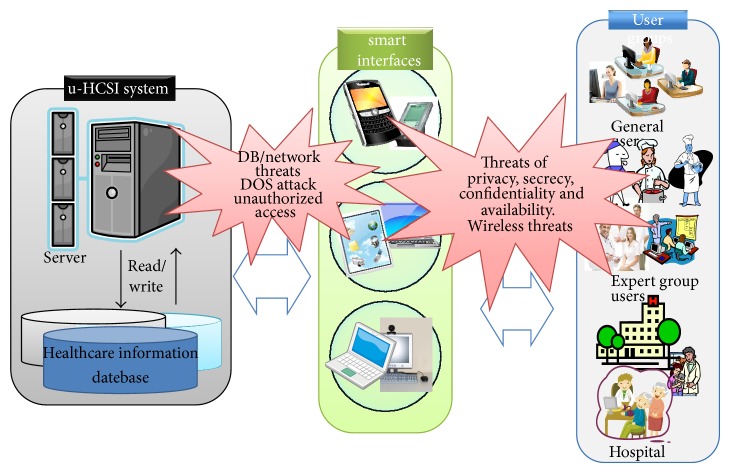
Security threats to u-healthcare service platform.

However, no perfect secure mechanisms exist in the healthcare applications, and sensitive privacy information can be leaked.  Figure 5 shows the security threats to u-healthcare service. As shown in Figure 5, traditional security threats such as DB/network vulnerabilities and unauthorized access can be potentially. In addition, more seriously, there exist the potential privacy leakage and threats to availability. Most healthcare information is classified as sensitive.

The details of the potential vulnerabilities are eavesdropping, denial of service, database security, network security, data protection, privacy protection, authentication, unauthorized access, message forgery, and virus attack.

As examining security threats in u-healthcare service, it is necessary to construct a security model including risk assessment, accountability and policy enforcement. 

*Risk Assessment.* The information asset can be a registry and repository, where the information of patient health is stored, it is guaranteed for data confidentiality, integrity, and availability.

*Accountability.* In addition, the patient safety is always the first to privacy is a primary principle of providing health information. The requester who try to access to the health information must be identified in order to determine the responsibility to impart information. In particular, a history of access control events with respect to the information must be sure to perform a security audit logs should be left.

*Policy Enforcement. *The domains which share information of each other have to agree with cross identification, authentication, and security audit level.

In this paper, to support both privacy protection and availability, we apply an RBAC. The RBAC security model is useful and flexible because of its usability, expressiveness, extension, and flexibility. In next section, we will describe the framework of u-HCSIP and construct an RBAC-based.

## 4. Framework of RBAC Based u-HCSIP 

### 4.1. Constructing RBAC Based Security Model

Personal healthcare services using telemedicine technology can be provided along with medical services and no restrictions in either time or location. Recent launches of Microsoft's “Health Vault” and “Google Health” are examples of a whole gamut of security risks compounding the privacy problem.

RBAC security model is useful and flexible. The primary motivation for RBAC lies in reducing the complexity and effort required to manage authorization data in large scale systems [7]. It has been designed for environments where discretionary access control (DAC) is inappropriate because end users do not own the information they are allowed to access and therefore should not have the discretion to grant access to others [5]. It is possible to construct a security policy for each enterprise using an RBAC. The basic concepts of role-based access control consist of user (U), role (R), sessions (S), and permission (P) [8]. In addition, role hierarchy, constraints, and privacy purpose group are included. Similar to the security management in the previous role of manager, functions of the security management, including creation of permission, roles, users, and role management, are subdivided. This model strengthens the function of reflecting policies, so that constraints and security controls are applied on all the components, along with the intensified role of manager, deriving flexibility from changes in the u-healthcare service environment.

Security management applied with RBAC simplifies the complicated procedures of granting authority in an extensive network environment, to reduce the required amount of time and costs for security management.

**Figure 6:**
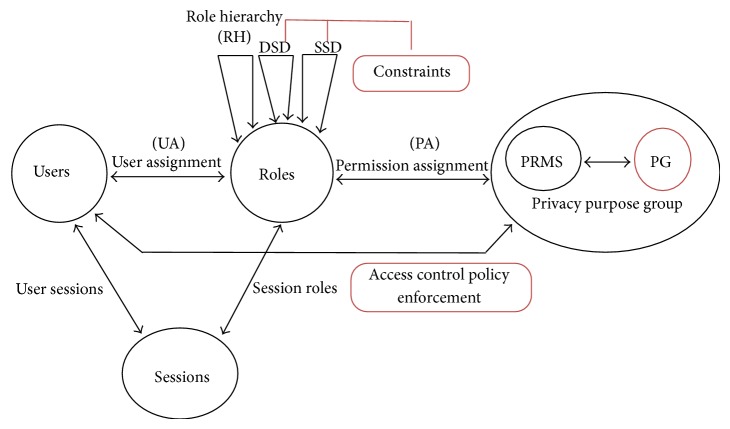
Extended RBAC security model.

Thus, it is more suitable than other access control models and can be applied to various environments. We extended general RBAC model because our u-HCSIP is used by many users with different security levels. So we appended PG (privacy purpose group) which was used to categorized user group according to security level that user had want to belong to. An extended RBAC security model is shown in Figure 6.

**Figure 7:**
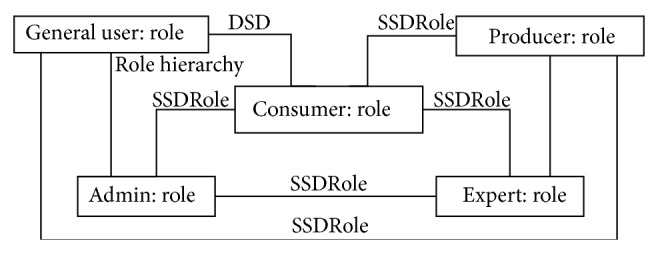
Object diagram for Extended RBAC security model in u-HCSIP.

Our security model can be used to specify a variety of constrains. In u-HCSIP user could have many roles. User who had roles of consumer also had a role of producer next time. Therefore there are many constraints of SSD (static separation of duty) and DSD (dynamic separation of duty). Object diagram of u-HCSIP was shown in Figure 7 and components of RBAC model were shown as follows:

 U (user): set of users,
 R (role): set of roles,
 (object): set of objects,
 P (permission): set of permissions (read, write, execute, append, delete, update),
 S (session): set of sessions,
 C (constraints): set of constraints,
 T (task): set of tasks, roles consisting of tasks,
 PG (purpose based group): set of purpose based groups, user can select his own privacy purpose group in u-HCSIP,
 SP (security policy): set of security policies,
 MR (manager role): set of manager roles,
 RH (role hierarchy): set of role hierarchies,
 UA: user assignment,
 PA: permission assignment,
 SA: session assignment,
 RA: roles assignment,
 TA: task assignment.

A security policy includes definitions of the subjects, objects, permissions, roles, role hierarchy, and constraints of an enterprise. The major benefits of an RBAC security model over other access control models are its usability and expressiveness.

Role engineering is what designs components of RBAC model, and properly constitutes policies in a particular organization.

Constraints of the duty separation are used to prevent conflicts with policies. Constraints of the SSD are intended to prevent conflicts that can occur when acquiring permission regarding the role against the user.

Constraints of the SSD-role using OCL (object constraint language) are presented as below.

 
   
*Constraints of SSD-Role: it is needed not assign conflicting role to the same user.*
 
***context*** *SSDRole.*
 
*Role_1.User *⟶* excludesAll (Role_2.User)*

Constraint of DSD is a restriction on the role to be promoted in the same user session. In other words, if a part of the role in DSD constraints is activated, the user is not able to activate other roles that conflict in the same session. Constraints of the DSD use OCL, and are represented as below.

 
   
*DSD constraint: it is needed not to activate conflicting role in the same session.*
 
***context*** *DSD*.
 
*Role_1.Session *⟶* excludesAll (Role_2.Session)*

The security policy expressiveness supports a practical requirement for efficient and manageable consent-based health information sharing.

### 4.2. The Framework of u-HCSIP

**Figure 8:**
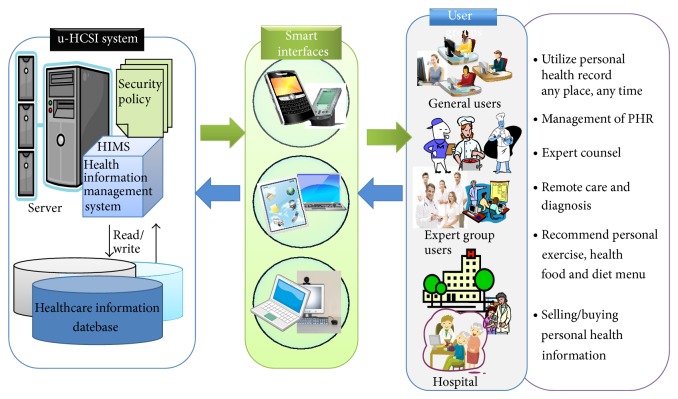
The framework of u-HCSIP (u-healthcare service integration platform).

In this section, we presented a framework of u-healthcare service platform applying RBAC.  Figure 8 showed the framework of u-HCSIP. u-HCSIP consisted of HIMS (health information managements system), SMS (security management system), health information database, and knowledge base. The main component HIMS carried out four key services. The first service is self-management of health data using a smart device. The second service is PHR management and exchanging service. The third service is the buying/selling personal health information. Finally, the fourth service is a drug information searching service.

A life-pattern service using the health care data of the proposed service platform is associated with information regarding food consumption, sleep, exercise, feeling, and the relevance of the disease. It is possible to recommend a service for diet and exercise by analyzing which foods change the user's bodily condition, using statistical information on food and exercise. In addition, personal health information, that is, PHRs, contains not only the user's physical characteristics, but also the information on their diseases and hospital medical records.

It has become recently possible to communicate messages according to the CCR standard based on XML. In the hospital, interacting with the hospital information system or Google Health will enable information exchange costs to be reduced by building CCR parsers and an information exchange system. The details of each service are as follows.

*(1) Information Management and Exchange Services. *The current hospital information system appears to have many problems in exchanging medical information among hospitals, including basic patient information; in addition, there is no standard format for medical records or hospital database design. HL7 is often used as the standard for medical information, but since it was designed substantially to match the U.S. health care system, there are many problems in its domestic application. The CCR standards currently being used by Google Health have defined the important information in this area. They are in a suitable form to represent medical record information and an individual's health status.

**Figure 9:**
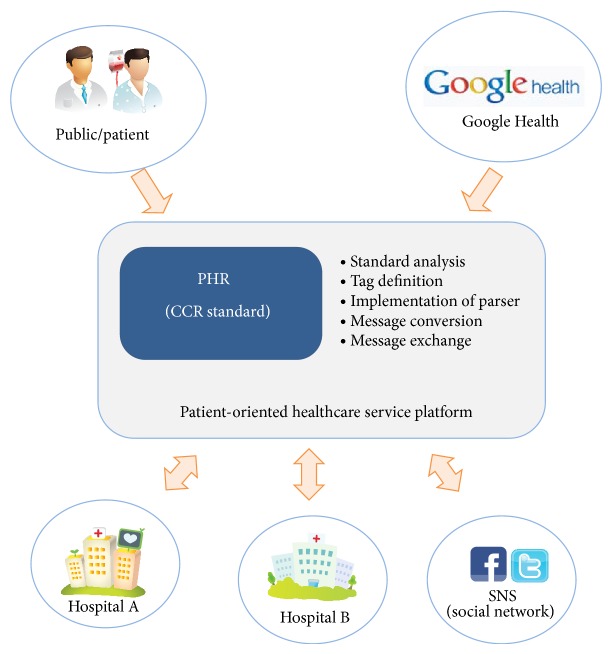
PHR exchange service diagram.

Seoul University Hospital, Aju University Hospital, and Gachon University's Gil Medical Center are researching and developing XML-based CCR standards. The personal smart healthcare service platform proposed in this paper saves data based on a CCR clinical information data, including the basic information of the patient, and adopts the transmission, reception, and conversion of messages.  Figure 9 shows the information management exchange service conceptual diagram of a PHR.

*(2) Health Data Self-Management Services Using a Smart Device: Health Log.* Obesity and chronic diseases such as high blood pressure and diabetes require daily management of food intake, impetus, and sleep. The proposed technique, as shown in Figure 9, records patient data in real time using a smartphone and then stores into the health information database personal health data regarding to user's actions and emotions in daily life in the server. Daily life recordings and statistics are also stored into the health information database in the server. It can be utilized to consult with an expert like doctor, fitness trainer and dietitian. Custom service in terms of the use patterns is also supported by utilizing data analysis techniques.

Health log service application using the smart device is to manage health related everyday life pattern data and to provide the customizing service of health information like food intake, meals, exercise and impetus to users. In order to provide personal customized information, HIMS (Health Information Managements System) performs data analysis using data mining techniques, machine learning, ontology based context awareness, inference rules, and pattern recognition. According to the frequency of occurrence of activities occurred in the day-to-day life, relationship, sequential and repetition relationship, analysis of personal health information is carried out by inference engine of the HIMS.

The differentiated services of the adequate support of the disease and health status for each user are (1) recommending evening meals to patients with lunar constitution of high blood pressure and diabetes, (2) daily caloric intake information considering the amount of food consumed and momentum, and (3) depression treatment information regarding the weather and states of emotional change.

*The User's Point of View.* Using the proposed health log application people who have chronic diseases or obesity can use smart devices anywhere, anytime and store behavior and emotional status data to the server. Applying health log app, self-diagnosis and monitoring is possible using the results of the analysis of the various statistics and patterns.

*Professional Perspective*. Experts (doctors, health managers, and nutritionists) provide professional knowledge on disease prediction and improved eating habits using day-to-day life data and pattern information of the users, as well as an adequate health management service upon regular admission to a hospital.

**Figure 10:**
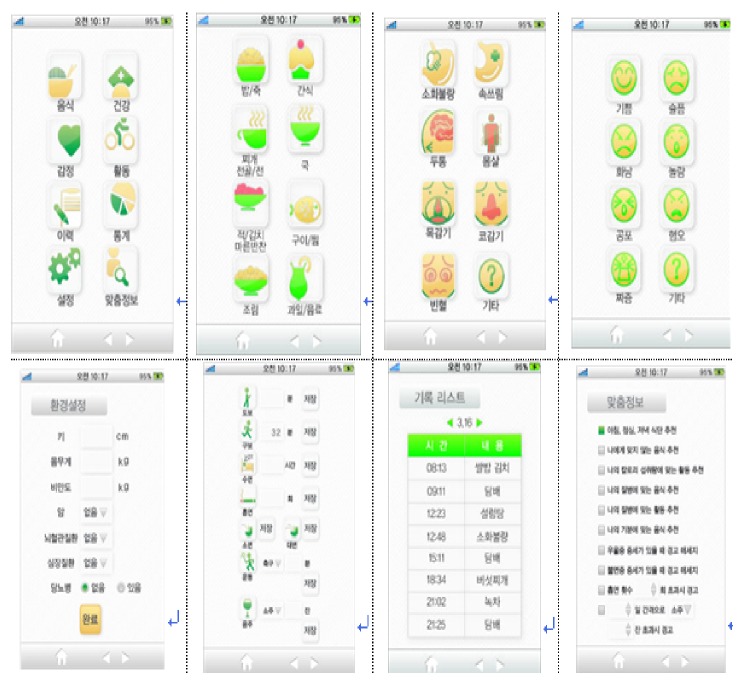
Smartphone application user interface.

Figure 10 shows an example UX interface of the smart phone application for health data self-management services. From the user's perspective, personal health related data can be entered easily using symbolic icons. In addition, a helpful service can be performed by utilizing useful knowledge obtained through the analysis of large amounts of data.

*(3) Buying and Selling Service Adequate Medical Information.* The Electronic Medical Record System is a method for computerizing all medical information in hospitals without changing any combination of IT technology to medical record management that had managed to paper charts existing.

Medical information like patient-specific health status, patient history, patient-specific treatment results, prescribed patient results, drug reactions by patients and patient's admission/discharge records are recorded in EMR formats. Most patients who were suffered from a specific illness want to get their own information about their disease and to handle it for the better treatment or any other way to overcome their disease. However, that kind of medical information cannot be provided to the patients or patients cannot receive information from other patient's medical information. To solve these problems, patient-oriented personalized smart healthcare services platforms configure the medical information database useful according to collective intelligence and search for medical information related to a buyer's request information with high similarity. Then personalized medical information sales service can be provided to the buyers who want to purchase medical information of other patient with similar conditions.

**Figure 11:**
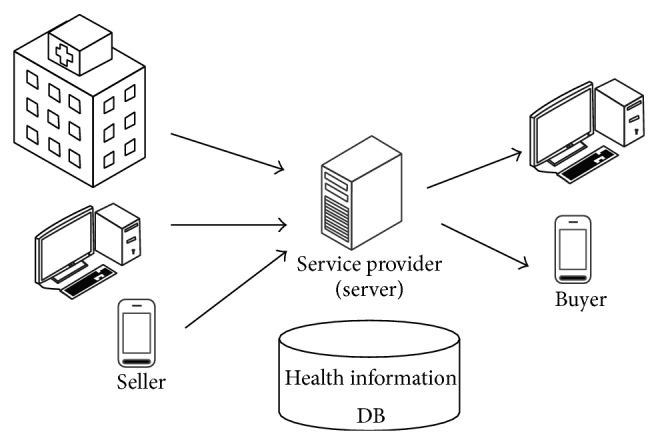
Buying/selling health information service.

Figure 11 shows a conceptual diagram of buying and selling services of adequate individual medical information. A service provider constitutes a medical information database. A service provider gradually stores information by building a medical information database provided from collective intelligence. In addition, a service provider maintains healthcare information database incrementally by adding health related data through on/off-line network.

The service provider provides a search capability of medical information of high similarity with a potential buyer's purchase information. In other words, if a buyer who was willing to buy medical information connects to the server providing the services and enters their purchase information, the server can support the service of searching medical information as depending on configured medical information database.

The method of sales service which provides personalized medical information is to search the health data related to purchase information registered by the seller. Similarity could be determined by the number of users who purchase information and when medical information matches. It was determined that the greater the number of matches, the higher the similarity. In addition, it will be able to extract as a result searched in case that purchase information and medical information are matching over a small number.

**Figure 12:**
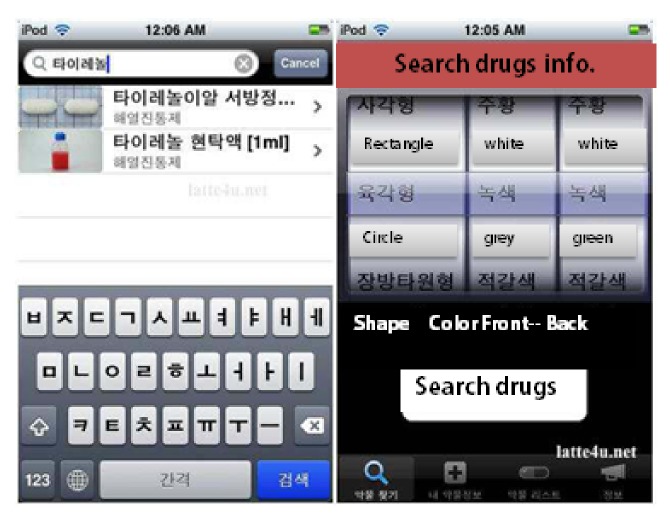
Search drug information.

*(4) Search Drug Information Services.* Finally, HIMS performs the useful search functions for drug information. Generally, patients cannot guess the efficacy or ingredients of a drug simply by seeing its form, even if the patient knows the prescribed drug; this includes drugs that have side effects. Searching and providing drug information services using smart devices can be divided into several steps. User scans and enters the shape of a drug, color of a drug and drug's name. Searching and providing drug information service can find out the drug related information as shown in Figure 12. So it can prevent the misuse or abuse of drugs, and that can prevent waste through the disposal of drugs using smart devices, has been provided.

### 4.3. Software Architecture of RBAC Based u-HCSIP

**Figure 13:**
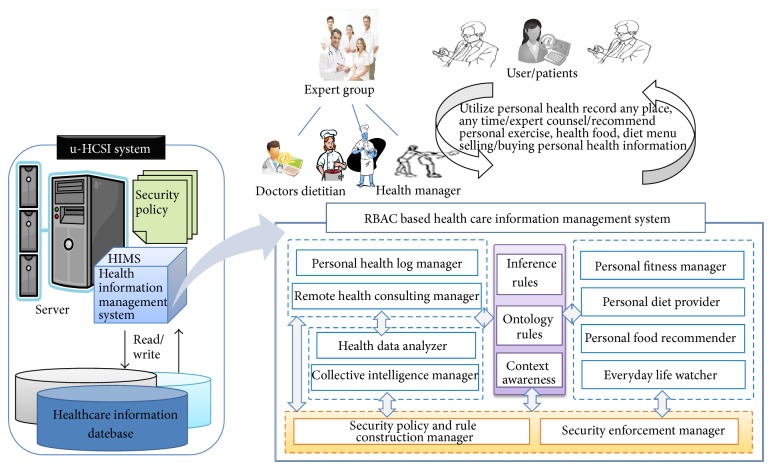
The software architecture of HIMS in u-HCSIP.

The design of the software architecture of the personalized smart healthcare service platform used to implement detailed functional specifications was shown in Figure 13.

The personalized smart healthcare service platform can be implemented applying many techniques such as medical information service platform technology, analytical techniques of personalized patient data, standardization technology of information exchanges, service development of exchange and management of PHR information, and application development for personalized health information management based on a smartphone. Management of remote consultation is a module for remote connections of general experts and users. Personalized health information services are a module that provides health considerations depending on the health status of the user. The personalized health information management stores and retrieves the PHRs of all personal. Providing an individual diet is a service module providing a personalized diet in consideration of personal physique, health, and favorite food for the public or those with chronic diseases. The personalized sports guidance service provides an appropriate exercise level, the kind of exercise, and exercise time in consideration of the person's height, weight, and age. A health information analyzer will be provided by extracting similar information to the health information required by buyers. It shows reliability based on the recommended amount of useful health information that has been purchased by many users.

The personalized health care system consisted of 8 functional components such as personal health log manager, personal remote consulting manager, personal diet provider, personal health data analyzer, collective intelligence manager, personal fitness manager, personal food guider, and everyday life watcher. In addition, there exists a security manager component which performed security policy enforcement.

Also an artificial agent, that constructs knowledge from the inference rules, context awareness rules and data mining rules, must be included in u-HCIS. In order to provide health information and to recommend appropriate health information, it analyzes the collective intelligence and mines useful information from the gathered health data.

All services are carried out with grant stamp of security enforcement server. First of all, health information consumer and provider gain a grant token to use the u-HCISP for the management of personal health records.

Using a smart device, a typical user can enter his information, receive the provisioning of a health information service, or receive the provisioning of consultation requests with a professional. In addition, health-related professionals in various fields, such as doctors, nutritionists, and fitness managers can provide consultation to a typical user, provide a variety of related information in terms of health, exercise, or food, or provide specific health warning information. Through a smart device, general users, and experts are connected to each other at a health care contact service.

If users have wanted to sell a variety of health information they had learned in their own experience, they can register with the health information to the service of buying/selling health information. Users who want to purchase the health information are able to buy by searching registered data such as health information that they are interested in.

**Figure 14:**
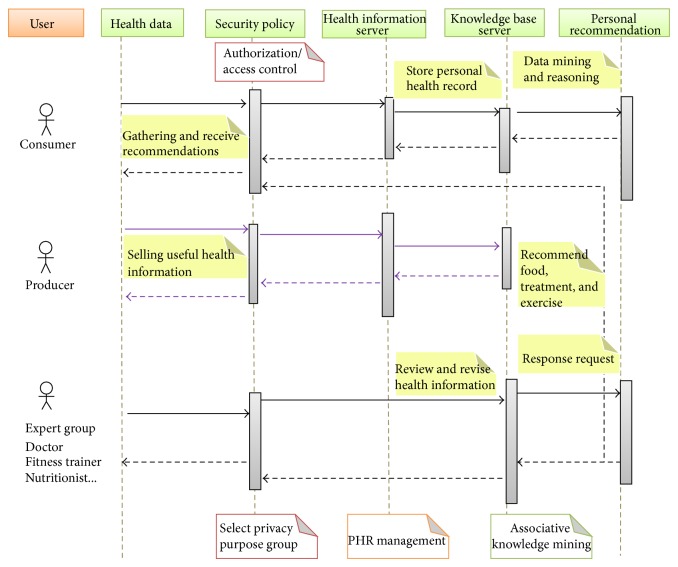
The workflow of u-HCSIP.

The user management module grants permission by distinguishing the user and applying an access control model of a role-based method for the protection of personal data, thereby protecting user information and user management for information related to personal privacy protection. The basic workflow of the healthcare service platform proposed in this paper is shown in Figure 14.

Figure 14 shows the use case and we can verify the proposed RBAC based u-HCISP works with guarantee of confidentiality and privacy. User of consumer role: gathers and manages health data with grant token of authentication and access control. Health information server carries out to update incremental database and to analyze reasoning rules. User of producer role: wants to sell one's own health information and registers health data with grant token of access control and authentication. The security service provider must classify user as a specific privacy group and assign the roles. Therefore user including consumer, producer, expert, can be handled to access control and privacy preserving.

## 5. Conclusions

E-healthcare informatics can be classified as sensitive information. Privacy is one of the key issues to be handled in e-healthcare informatics.

In this paper, we examined security requirements related to privacy protection and extended RBAC security model.

Then we proposed RBAC-based personalized healthcare service platform for the smart management of personal health records using smart devices. We have designed u-healthcare service integration platform (u-HCSIP) and applied RBAC based security model in order to support secure u-healthcare service. The proposed RBAC based u-HCISP can have performed four main functions: storing and exchanging personal health records (PHR), meals and exercise recommendations, buying/selling private health information or experience, and search information of drugs service. We have verified the usefulness of the proposed RBAC-based u-HCSIP by analyzing the use case workflow.

The proposed RBAC-based u-HCSIP is useful not only for smart mobile healthcare management but also for improving the quality of life in the coming aging society.

## Figures and Tables

**Table 1:** u-healthcare trend of global companies.

Global company	u-healthcare trend
IBM	Remote monitoring and personal health measurement u-healthcare solutions offering

Microsoft	Development of searching and sharing medical information system

Philips	Release health management services (Moriva) that are customized using the TV for elderly who are not familiar with the use of the Internet

Intel	Release the digital health service areas such as home-based medical care and computerized hospital. Releasing medical mobile devices with built-in RFID reader MCAP (mobile clinical assistant platform)

Qualcomm	Establish LifeWatch, heart monitoring, wireless communication, GPS, real-time response for u-healthcare mobile service provider
